# Expression of genes associated with immunity in the endometrium of cattle with disparate postpartum uterine disease and fertility

**DOI:** 10.1186/1477-7827-7-55

**Published:** 2009-05-29

**Authors:** Shan Herath, Sonia T Lilly, Natalia R Santos, Robert O Gilbert, Leopold Goetze, Clare E Bryant, John O White, James Cronin, I Martin Sheldon

**Affiliations:** 1 Department of Veterinary Clinical Sciences, Royal Veterinary College, University of London, Royal College Street, London, NW1 0TU, UK; 2 Department of Clinical Sciences, College of Veterinary Medicine, Cornell University, Ithaca, New York 14853, USA; 3 Pfizer Animal Health Europe, 23–25, avenue du Dr Lannelongue, F-75668, Paris Cedex 14, France; 4 Department of Veterinary Medicine, University of Cambridge, Madingley Road, Cambridge, CB3 0ES, UK; 5 Institute of Life Science, School of Medicine, Swansea University, Singleton Park, Swansea, SA2 8PP, UK

## Abstract

**Background:**

Contamination of the uterine lumen with bacteria is ubiquitous in cattle after parturition. Some animals develop endometritis and have reduced fertility but others have no uterine disease and readily conceive. The present study tested the hypothesis that postpartum cattle that develop persistent endometritis and infertility are unable to limit the inflammatory response to uterine bacterial infection.

**Methods:**

Endometrial biopsies were collected several times during the postpartum period from animals that were subsequently infertile with persistent endometritis (n = 4) or had no clinical disease and conceived to first insemination (n = 4). Quantitative PCR was used to determine the expression of candidate genes in the endometrial biopsies, including the Toll-like receptor (TLR 1 to 10) family of innate immune receptors, inflammatory mediators and their cognate receptors. Selected proteins were examined by immunohistochemistry.

**Results:**

The expression of genes encoding pro-inflammatory mediators such as interleukins (IL1A, IL1B and IL6), and nitric oxide synthase 2 (NOS2) were higher during the first week post partum than subsequently. During the first week post partum, there was higher gene expression in infertile than fertile animals of TLR4, the receptor for bacterial lipopolysaccharide, and the pro-inflammatory cytokines IL1A and IL1B, and their receptor IL1R2. The expression of genes encoding other Toll-like receptors, transforming growth factor beta receptor 1 (TGFBR1) or prostaglandin E_2 _receptors (PTGER2 and PTGER4) did not differ significantly between the animal groups. Gene expression did not differ significantly between infertile and fertile animals after the first week postpartum. However, there were higher ratios of IL1A or IL1B mRNA to the anti-inflammatory cytokine IL10, during the first week post partum in the infertile than fertile animals, and the protein products of these genes were mainly localised to the epithelium of the endometrium.

**Conclusion:**

Cattle may maintain fertility by limiting the inflammatory response to postpartum bacterial infection in the endometrium during the first week after parturition.

## Background

Contamination of the uterine lumen with bacteria affects 90 to 100% of dairy cattle within the first week after parturition [1-3]. After infection with bacteria, some cattle have an appropriate immune response, eliminate the bacteria efficiently and have optimal fertility but 25 to 30% of animals have a florid and persistent inflammatory response in the endometrium (endometritis), and are often infertile [3,4].

The endometrium is the first line of defence against bacteria that ascend the female genital tract after parturition. Beyond a barrier function, the endometrial cells have important roles in innate immune defence in cattle, humans and rodents [5-7]. The initial defence of the endometrium against microbes is dependent on innate immune systems including Toll-like receptors and antimicrobial peptides [8,9]. The Toll-like receptors (TLRs) recognise pathogen associated molecular patterns, and ten members of the receptor family are widely encoded in the mammalian genome [10,11]. Briefly, TLR1, TLR2, and TLR6 recognise bacterial lipids such as lipoteichoic acid (LTA), whereas TLR3, TLR7, TLR8, and TLR9 recognize nucleic acids, often from viruses. TLR4 recognizes lipopolysaccharide (LPS) from Gram-negative bacteria such as *Escherichia coli*. TLR5 binds bacterial flagellin, and TLR9 also recognises bacterial DNA. The nucleotide-binding oligomerization domain (NOD) receptors (NOD1 and NOD2) detect bacteria that have invaded inside host cells. The LPS/TLR4 pathway is functional in the bovine endometrium [5,12], and the other nine TLRs appear to be expressed at the mRNA level [13].

Engagement of TLRs results in the production of pro-inflammatory mediators including cytokines and chemokines that direct the immune response to prevent propagation of the pathogens and eliminate them from the tissues [10,11,14]. For example, the cytokines interleukin-1 (IL-1), IL-6 and tumour necrosis factor (TNF) stimulate the production of anti-microbial peptides to help eradicate the pathogenic bacteria. However, the duration and magnitude of the immune response has to be regulated to avoid persistent tissue inflammation [15]. Pro-inflammatory cytokines are regulated by negative feed-back loops and the production of anti-inflammatory cytokines such as IL-10 [14,16]. Prostaglandin E_2 _also limits the inflammatory response by acting through the prostaglandin E receptors, EP2 and EP4 that are encoded by the genes PTGER2 and PTGER4, respectively [17]. Some cattle appear to have an appropriate immune response, clear the bacteria, and the endometrium returns to normal. Other animals have persistent chronic inflammation of the endometrium and fertility is compromised [3,4]. The role of the chemokine IL-8 in endometritis has been widely investigated but the cytokine response is less clear [18]. Pro-inflammatory cytokines such as TNF and IL-1 may also perturb fertility by interfering with the production or action of hormones in the endometrium and ovaries of cattle [19-21].

The present study tested the hypothesis that postpartum cattle that develop persistent endometritis and infertility are unable to limit the magnitude or duration of the inflammatory response to uterine bacterial infection. The objective was to use quantitative PCR to compare the expression of candidate genes involved in innate immune recognition of pathogens and the inflammatory response, particularly cytokines, in endometrial biopsies from infertile animals with persistent endometritis and animals with no clinical disease that conceived to first insemination.

## Methods

### Animals and sample collection

Animals were from a population of approximately 80 Holstein cows in a dairy herd at Cornell University with a mean annual milk production of 12,000 kg per cow. The cows were housed in a tie-stall barn, bedded on straw that was cleaned three times per day, and the cows had access to an exercise pen every day for 3 h. The cows were fed a total mixed ration formulated to meet or exceed the National Research Council (2001) nutrient requirements for lactating Holstein cows weighing 680 kg and producing 45 kg of 3.5% fat corrected milk. The cows included in the study were evaluated at parturition and weeks 1, 3, 5 and 7 post partum. All procedures were approved by the Cornell University Institutional Animal Care and Use Committee (Protocol No. 2004-0078). Fertility data, including uterine disease, the dates of artificial insemination and pregnancy diagnosis were recorded for at least 200 days after calving for each animal.

At parturition and 1, 3, 5 and 7 weeks post partum, the cows were clinically examined and sampled by uterine biopsy and fluid collection. Clinical uterine disease was diagnosed using the accepted definitions by examination of the contents of the vagina and rectal palpation of the genital tract three times per week as previously described [3,4,22]. Briefly, cows were diagnosed with puerperal metritis if they had a fetid uterine discharge, flaccid uterus and fever (> 39.5°C), with clinical endometritis if purulent or mucopurulent vaginal discharge was evident on manipulation of the genital tract 3 weeks post partum, and with subclinical endometritis on the basis of endometrial cytology samples containing more than 10% neutrophils after 35 days post partum. A total of 28 cows had complete data and sample sets during the study and from these, animals were retrospectively selected for investigation of gene expression based on disparate uterine disease and fertility. The first group ("infertile", n = 4) comprised animals that had persistent metritis, clinical endometritis and later subclinical endometritis, and did not conceive to at least 3 inseminations within 200 days of parturition. The second group ("fertile", n = 4) showed no signs of uterine disease and conceived at the time of first insemination (range 59 to 74 days in milk).

Endometrial biopsies were collected using an endometrial biopsy instrument (Hauptner, Solingen, Germany). The biopsy instrument was protected by a sanitary chemise and passed through the cervix guided by trans-rectal palpation to the site of biopsy at the level of the bifurcation of the uterine horns. Once the instrument was in place an 8 × 5 mm piece of tissue was collected, the instrument was withdrawn, the tissue placed immediately into a 1.5 ml tube containing a preservative to maintain mRNA integrity (RNAlater; Qiagen, Crawley, U.K.), and a second biopsy was formalin-fixed and paraffin-embedded for immunohistochemistry. Uterine fluid samples were obtained by uterine flush as previously described [4]. Briefly, the vulva lips were cleaned with dry paper and an intrauterine pipette protected by a sanitary chemise was introduced through the vagina and cervix into the uterus, guided by transrectal palpation. The chemise was ruptured and 20 ml sterile lactate ringer solution infused into the uterine lumen, retrieved and placed in a sterile collection tube. A swab of this fluid was transferred using the Port-A-Cul (PAC) transport system (BD, Franklin Lakes, New Jersey, USA) to the Cornell University Animal Health Diagnostic Centre for anaerobic and aerobic culture, and identification of bacterial isolates using standard procedures [22].

### RNA isolation and reverse transcription

Total RNA was isolated from 20 to 70 mg of endometrial tissue using the RNeasy Midi Kit (Qiagen) and the concentration and purity of the RNA samples was determined using a NanoDrop^® ^(ND-1000 Spectrophotometer, NanoDrop Technologies Inc, Delaware, USA). All samples had an A_260/280 _absorbance ratio of between 1.85 and 2.0. Following quantification, all sample RNA was normalised to100 ng/μL, DNase treated (Promega, Southampton, U.K.) and reverse transcribed into First Strand cDNA using SuperScript II RNase H^- ^Reverse Transcriptase (Invitrogen, Life Technologies, Paisley, U.K.) according to the manufacturers' protocols.

### Optimisation of RT-PCR

To optimise PCR conditions for each gene, conventional PCR was employed using 60–70 ng of cDNA, GoTaq^® ^Green Master Mix (Promega) and primers (20 pM). Intron-spanning gene-specific primers that were short enough to ensure optimum amplification were designed using sequences published in the National Center for Biotechnology Information database (Bethesda, Maryland, USA) using Primer3 software [23], and purchased from MWG (Eurofins MWG Operon, Ebesberg, Germany). Following optimisation, the presence of a single product was confirmed on a 1.5% agarose gel by electrophoresis and products were sequenced in house using an ABI 3100 genetic analyzer and Bigdye Terminator 3.1 from ABI (Foster City, California, USA) and shown to be 92 to 100% homologous to the BLAST database sequences. Primer pair sequences along with optimised annealing temperatures are presented in Table 1 or in Davies et al [13].

**Table 1:** Oligonucleotide primer sequences.

Gene		Primer sequence	Size	Tm (°C)	Accession No.
*CD45*	Sense	CTCGATGTTAAGCGAGAGGAAT	185	56	AJ400864
	Anti sense	TCTTCATCTTCCACGCAGTCTA			
*CD14*	Sense	GGGTACTCTCTGCTCAAGGAAC	199	56	NM_174008
	Anti sense	CTTGGGCAATGTTCAGCAC			
*MD-2*	Sense	GGGAAGCCGTGGAATACTCTAT	204	54	DQ319076
	Anti sense	CCCCTGAAGGAGAATTGTATTG			
*NOD1*	Sense	GTCACTCACATCCGAAACACTC	213	55	XM_598513
	Anti sense	CCTGAGATCCACATAAGCGTCT			
*IL1A*	Sense	AGAGGATTCTCAGCTTCCTGTG	224	54	NM_174092
	Anti sense	ATTTTTCTTGCTTTGTGGCAAT			
*IL1B*	Sense	GAGGAGCATCCTTTCATTCATC	229	56	X54796
	Anti sense	TTCCTCTCCTTGTACGAAGCTC			
*IL1R2*	Sense	ATCCCATGTAAGGTGTTTCTGG	181	56	AB219098
	Anti sense	TGACAGGATCAAAAATCAGTGG			
*TNF*	Sense	ACTCAGGTCCTCTTCTCAAGCC	774	56	NM_173966
	Anti sense	ATGATCCCAAAGTAGACCTGCC			
*IL6*	Sense	ATGACTTCTGCTTTCCCTACCC	180	56	NM_173923
	Anti sense	GCTGCTTTCACACTCATCATTC			
*IFN-a*	Sense	AGAGCCTCCTGGACAAGCTAC	212	56	NM_001017411
	Anti sense	CATGACTTCTGCTCTGACAACC			
*NOS2*	Sense	GGACAGTAAAGACGTCTCCAGA	197	54	AF340236
	Anti sense	TATGGTCAAACTTTTGGGGTTC			
*IL10*	Sense	TACTCTGTTGCCTGGTCTTCCT	178	56	NM_174088
	Anti sense	AGTAAGCTGTGCAGTTGGTCCT			
*TGFBR1*	Sense	CAGGTTTACCATTGCTTGTTCA	243	56	NM_174621
	Anti sense	TGCCATTGTCTTTATTGTCTGC			
*PTGER2*	Sense	GTTCCACGTGTTGGTGACAG	246	56	AF539402
	Anti sense	ACTCGGCGCTGGTAGAAGTA			
*PTGER4*	Sense	TCGTGGTGCTCTGTAAATCG	226	56	AF539403
	Anti sense	CTCATCGCACAGATGATGCT			

Oligonucleotide primer sequences, expected amplicon size and annealing temperatures used in real-time PCR assays.

### Quantitative PCR

The cDNA was quantified against standards rather than making a comparison to a housekeeping gene because endometrial samples came from different animals and with the potential for variation in cellular composition of each biopsy, which might affect the level of expression of housekeeping genes. External standards for quantification of cDNA were prepared from each gene product. Each gene product was purified using the QIAquick PCR purification kit (Qiagen) and the precise concentration of cDNA determined using the NanoDrop ND-1000 Spectrophotometer (NanoDrop Technologies Inc). Standards were prepared by serial dilution in nuclease-free water and ranged from 3 × 10^5 ^fg/μL to 3 × 10^-13 ^fg/μL. Gene transcripts were quantified for the endometrial biopsies by real-time PCR using the DNA Engine Opticon 2 (Bio-Rad, Hercules, CA, USA). A mastermix was prepared for each assay containing 2× SYBR green PCR mix (Sigma, Poole, UK), 800 nM forward and reverse primer and nuclease-free water. For each sample and gene transcript, a 25 μL reaction volume of mastermix containing 75 ng of cDNA was prepared into each well of a white 96-well plate. To ensure reproducibility and reduce variability, allowing for statistical analysis across the assay, all samples were run on a single plate and external standards and a blank control were run on each plate in duplicate. Cycling conditions consisted of an initial activation step of 95°C for 15 min followed by 39 cycles of denaturation, annealing, extension and fluorescence acquisition reading. To prevent acquisition of smaller non-specific products, a melting curve analysis was performed. All results were recorded and analysed using the Opticon Monitor Analysis Software (V2.02; Bio-Rad).

### Immunohistochemistry

To confirm the presence of selected proteins and their localisation immunohistochemistry was performed on the formalin-fixed paraffin-embedded endometrial biopsies from the postpartum cows. Briefly, 5 μm thick sections were deparaffinised, rehydrated, and antigen retrieval was performed using Tris- ethylenediaminetetraacetic acid (EDTA) buffer (10 mM Tris Base, 1 mM EDTA solution, 0.05% Tween 20, pH 9.0). Sections were then permeabilised in Tris-buffered saline (TBS) 0.025% Triton-X (Sigma) with gentle agitation and blocked for 2 h in 5% Donkey serum (Jackson ImmunoResearch Laboratories Inc, Pennsylvania, USA) with 1% bovine serum albumin (BSA; Sigma) in TBS. Sections were incubated overnight with the following primary antibodies at a dilution of 1:100: Rabbit IgG TLR4 (a kind gift from H-M Seyfert); rabbit IgG IL-6 (AHP424; AbD serotec, Raleigh, North Carolina, USA); rabbit IgG TNF (PBOTNFA1; Endogen, Cambridge, Massachusetts, USA); rabbit IgG IL-10 (ab34843; Abcam, Cambridge, Massachusetts, USA) mouse IgG IL-1 alpha (P420A; Thermo Fisher, Pittsburg, Pennsylvania, USA), mouse IgG IL-1 beta (PBOIL1B1; Thermo Fisher) and mouse IgG cytokeratin (6401-100; Abcam, Cambridge, Massachusetts, USA). An isotype control was included in each case. Subsequently the slides were washed twice in TBS 0.025% Triton-X for 5 mins. Either a donkey anti-mouse or a donkey anti-rabbit Alexa Fluor 555 labelled secondary antibody (Invitrogen) was then applied and the slides incubated in the dark for 1 h. The slides were washed three times in TBS for 5 min, mounted using Vectashield (Vector Labs, Burlingame, California) containing 4',6-diamidino-2-phenylindole (DAPI) and examined using an epifluorescent microscope (Axio Imager.M1; Zeiss, Jena, Germany) and images captured using a digital camera and appropriate software (Zeiss).

### Statistical analysis

The gene expression data were divided into two time periods, representing calving to 7 days post partum ("Period 1": 0 and 1 week post partum samples) or > 7 days ("Period 2": 3, 5 and 7 week post partum samples). The 7-day cut-off was selected because the magnitude of bacterial infection in the first 7 days postpartum is prognostic for the subsequent severity of uterine disease and infertility [1,22,24]. Analysis was done using the SPSS computer program (SPSS Inc., Chicago, Illinois, USA). Comparisons of gene expression between fertile and infertile animals, or between time periods, were tested using the non-parametric Mann-Whitney test. Results are reported as the arithmetic mean ± S.E.M., and significance ascribed when P < 0.05.

## Results

As expected, the animals with uterine disease had more bacterial isolates than the fertile animals (median 4.5 vs 1.0 isolates; P < 0.05) during the postpartum period. *E. coli *was isolated from 2 fertile and 3 infertile animals in Period 1 but not in Period 2, whereas *Arcanobacterium pyogenes *was isolated from 1 fertile and 3 infertile animals in Period 1, and from the same animals in Period 2. Anaerobic bacteria were isolated from none of the fertile animals in either Period but 2 infertile animals in Period 1 (1 *Prevotella *and 1 *Bacteroides *species), and 4 animals in Period 2 (1 *Prevotella*, 2 *Bacteroides *species, and 1 *Fusobacterium necrophorum*). *Ureaplasma*, *Haemophilus somnus *and *Streptococci *were also occasionally isolated from the uterine lumen.

### Immune detectors

**Figure 1:**
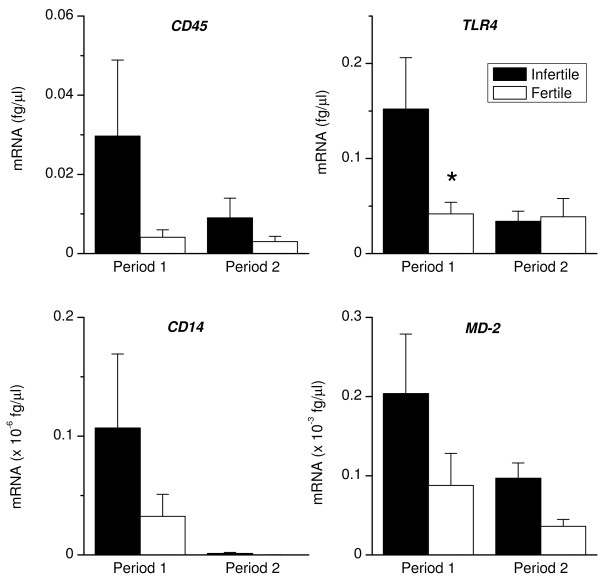
**Endometrial expression of *CD45, TLR4, CD14*, and *MD-2***. Expression of mRNA encoding *CD45, TLR4, CD14*, and *MD-2 *in endometrial biopsies collected from infertile (closed bar) and fertile animals (open bar), during Periods 1 and 2. RNA was isolated from biopsies, reverse transcribed and analysed by quantitative PCR for the mRNA encoding pan-leukocyte marker, *CD45 *and components of the LPS receptor complex, *TLR4, CD14 *and *MD-2*. *P < 0.05 compared with infertile animals, within the period. Numerical values are presented as the mean + SEM.

The ability of animals to clear infection is dependent largely on the ability to recognise pathogens and respond accordingly [10,11,14]. To determine whether there was a difference in this ability, we analysed the mRNA encoding the pan-leukocyte marker, *CD45 *to assess the infiltration of professional immune cells into the uterus. Infertile animals had greater *CD45 *expression during Period 1 than Period 2 (P < 0.05; Fig. 1), and infertile animals tended to have a higher level of expression of *CD45 *during Period 1 than fertile animals (P = 0.10). The expression of *TLR4, CD14 *and *MD-2 *(encoding MD-2 protein; alternatively known as *LY96 *encoding Lymphocyte antigen 96 protein) was examined next because *E. coli *infection is particularly important after parturition and paves the way for other pathogens to cause uterine disease [1,24]. Infertile animals had a higher level of *TLR4 *expression than fertile cattle during Period 1 (Fig. 1). The level of gene transcript expression for *TLR4, CD14 *and *MD-2 *decreased in infertile animals between Periods 1 and 2 (P < 0.05), although there was no significant difference in gene expression between the two groups of animals within Period 2 (Fig. 1). Fertile animals had similar levels of expression of *CD45, TLR4, CD14 *and *MD-2 *in Periods 1 and 2.

**Figure 2:**
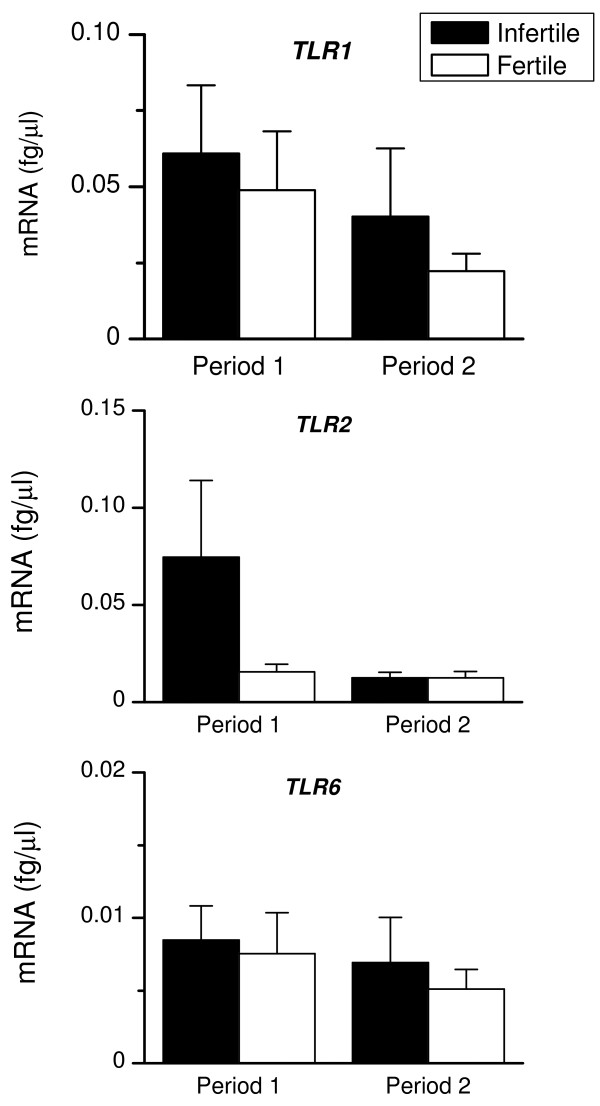
**Endometrial expression of *TLR1, TLR2 *and *TLR6***. Expression of mRNA encoding *TLR1, TLR2 *and *TLR*6 in endometrial biopsies collected from infertile (closed bar) and fertile animals (open bar), during Periods 1 and 2. RNA was isolated from biopsies, reverse transcribed and analysed by quantitative PCR for the mRNA encoding bacterial lipoprotein receptors, *TLR1, TLR2 *and *TLR6*. Numerical values are presented as the mean + SEM.

**Figure 3:**
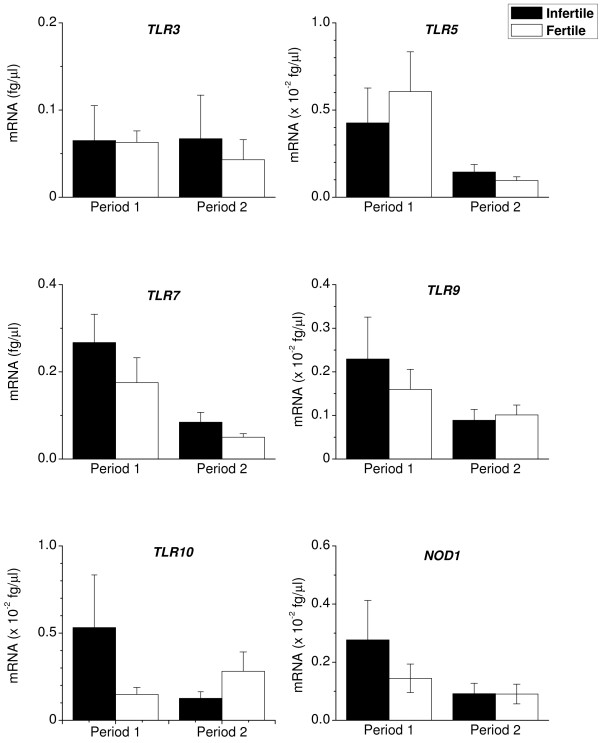
**Endometrial expression of *TLR3, TLR5, TLR7, TLR9, TLR10 *and *NOD1***. Expression of mRNA encoding *TLR3, TLR5, TLR7, TLR9, TLR10 *and *NOD1 *in endometrial biopsies collected from infertile (closed bar) and fertile animals (open bar), during Periods 1 and 2. RNA was isolated from biopsies, reverse transcribed and analysed by quantitative PCR for the mRNA encoding intracellular receptors *TLR3, TLR7, TLR9 *and *NOD1*, for the flagellin receptor *TLR5*, and for *TLR10 *that has unknown function. Numerical values are presented as the mean + SEM.

Both groups of animals expressed *TLR1, TLR2, TLR6 *(Fig. 2) and *TLR3, TLR5, TLR7, TLR9, TLR10 *and *NOD1 *(Fig. 3) in Period 1. There tended (P = 0.07) to be higher levels of *TLR2 *and *TLR10 *in endometrial biopsies from infertile than fertile animals during Period 1. The level of expression of *TLR2 *decreased between Periods 1 and 2 in the infertile animals (P < 0.05; Fig. 2). In Period 2, both fertile and infertile animals expressed similar levels of the *TLRs 1 *to *10 *and *NOD1*.

### Immune responses

**Figure 4:**
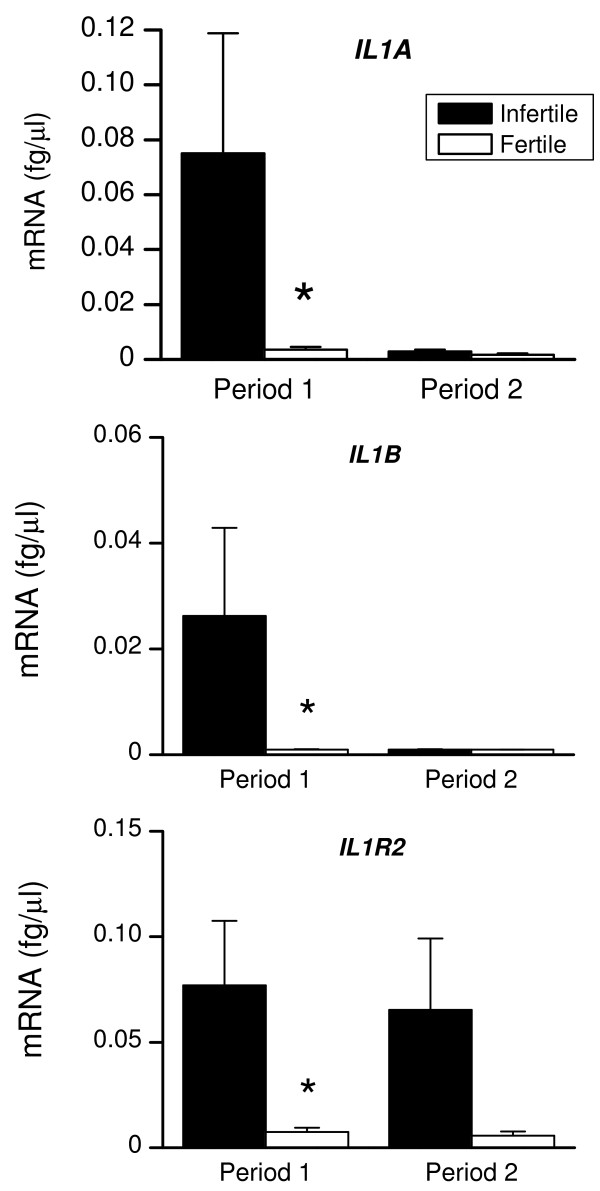
**Endometrial expression of *IL1A, IL1B *and *IL1R2***. Expression of mRNA encoding *IL1A, IL1B *and *IL1R2 *in endometrial biopsies collected from infertile (closed bar) and fertile animals (open bar), during Periods 1 and 2. RNA was isolated from biopsies, reverse transcribed and analysed by quantitative PCR for the mRNA encoding the pro-inflammatory cytokine isoforms *IL1A *and *IL1B *and corresponding receptor, *IL1R2*. *P < 0.05 compared with infertile animals, within the Period. Numerical values are presented as the mean + SEM.

**Figure 5:**
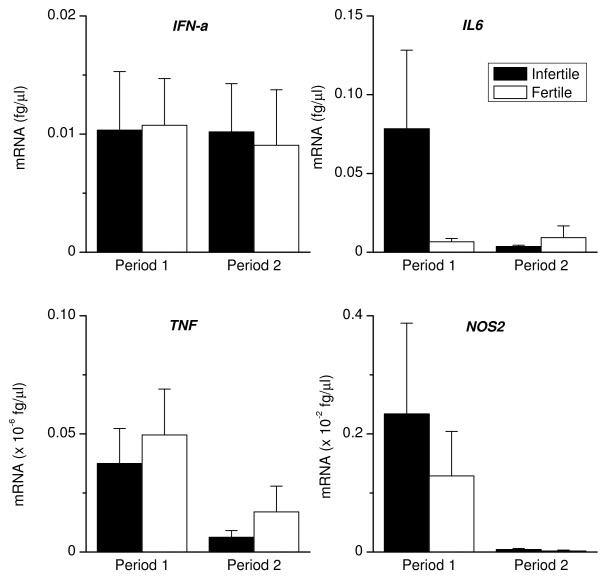
**Endometrial expression of *IFN-a, IL6, TNF *and *NOS2***. Expression of mRNA encoding *IFN-a, IL6, TNF *and *NOS2 *in endometrial biopsies collected from infertile (closed bar) and fertile animals (open bar), during Periods 1 and 2. RNA was isolated from biopsies, reverse transcribed and analysed by quantitative PCR for the mRNA encoding the pro-inflammatory mediators *IFN-a, IL6, TNF *and *NOS2*. Numerical values are presented as the mean + SEM.

To determine whether the animals with disparate disease and fertility outcomes mounted different immune responses to infection, we analysed endometrial tissue for mRNA encoding immune mediators. Figure 4 shows that during Period 1, there was a higher level of expression of *IL1A, IL1B *and *IL1R2*, in infertile than fertile animals. During Period 2, there also tended to be a higher expression of *IL1B *(P = 0.06) in infertile than fertile animals. Between Periods 1 and 2, the expression of *IL1A *decreased (P < 0.05) in infertile animals, and *IL1B *decreased (P < 0.05) in fertile animals. The levels of *IFN-a, TNF *and *NOS2 *(Fig. 5) were similar for fertile and infertile animals during Period 1, although *IL6 *tended to be higher in infertile animals (P = 0.06). There was no difference in the level of expression of the immune mediators between the two groups of animals during Period 2. Between Period 1 and 2, there was a decrease in the level of expression of *IL6 *(P < 0.05) and *NOS2 *(P < 0.05) in infertile animals and *NOS2 *(P < 0.05) for fertile animals.

### Anti-inflammatory versus pro-inflammatory responses

**Figure 6:**
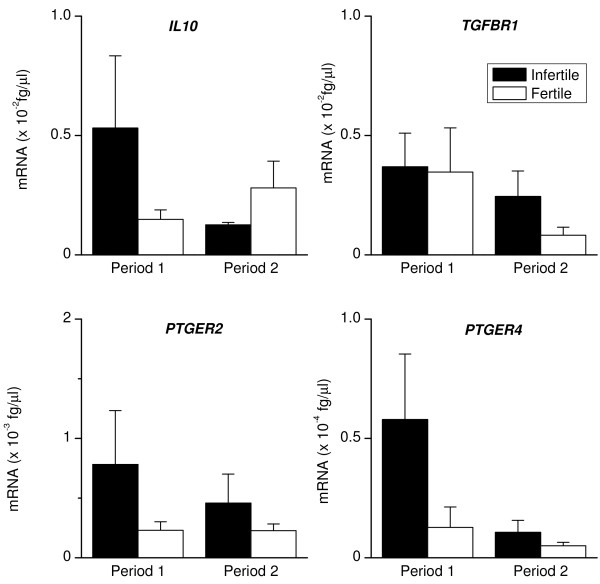
**Endometrial expression of *IL10, TGFBR1, PTGER2 *and *PTGER4***. Expression of mRNA encoding *IL10, TGFBR1, PTGER2 *and *PTGER4 *in endometrial biopsies collected from infertile (closed bar) and fertile animals (open bar), during Periods 1 and 2. RNA was isolated from biopsies, reverse transcribed and analysed by quantitative PCR for the mRNA encoding the anti-inflammatory mediator *IL10*, and the receptors for the anti-inflammatory mediators transforming growth factor beta 1 (*TGFBR1*) and prostaglandin E_2 _(*PTGER2 *and *PTGER4*). Numerical values are presented as the mean + SEM.

**Figure 7:**
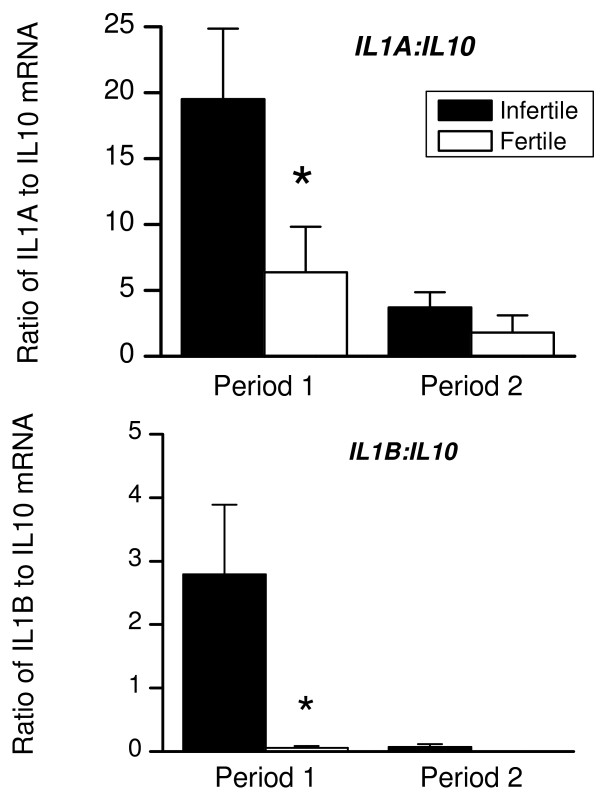
**Ratio of *IL1A *and *IL1B *to *IL10 *expression**. Ratio of expression of mRNA encoding *IL1A *or *IL1B *to *IL10*. The relationship between the expression of mRNA encoding *IL1A *or *IL1B *to *IL10 *was determined for infertile (closed bars) and fertile animals (open bars) using numerical values obtained by Q-PCR.

Both groups of animals expressed mRNA encoding the anti-inflammatory mediator *IL10 *(Fig. 6), although there was no difference in the level of expression between the two groups or the two Periods. Prostaglandin E_2 _and transforming growth factor beta 1 are also important anti-inflammatory mediators secreted by endometrial cells acting via EP2 or EP4 and transforming growth factor beta 1 receptor (TGFBR1), respectively [12,17,25,26]. During Period 1, *PTGER2 *and *PTGER4 *expression did not differ significantly between infertile and fertile animals, although the level of *PTGER4 *expression decreased (Fig. 6, P < 0.05) between Period 1 and 2 for the infertile animals. There were no significant differences in TGFBR1 expression between the animal groups or Periods (Fig. 6). Disease outcome is often dependent on the balance of pro-inflammatory and anti-inflammatory cytokines produced during an immune response such as IL-1 and IL-10, respectively. Figure 7 shows that during Period 1 there was a higher ratio of *IL1A *or *IL1B *to *IL10 *in infertile than fertile animals. The ratio of *IL1A *or *IL1B *to *IL10 *decreased between Period 1 and 2 in infertile animals (P < 0.05).

### Immunohistochemistry

**Figure 8:**
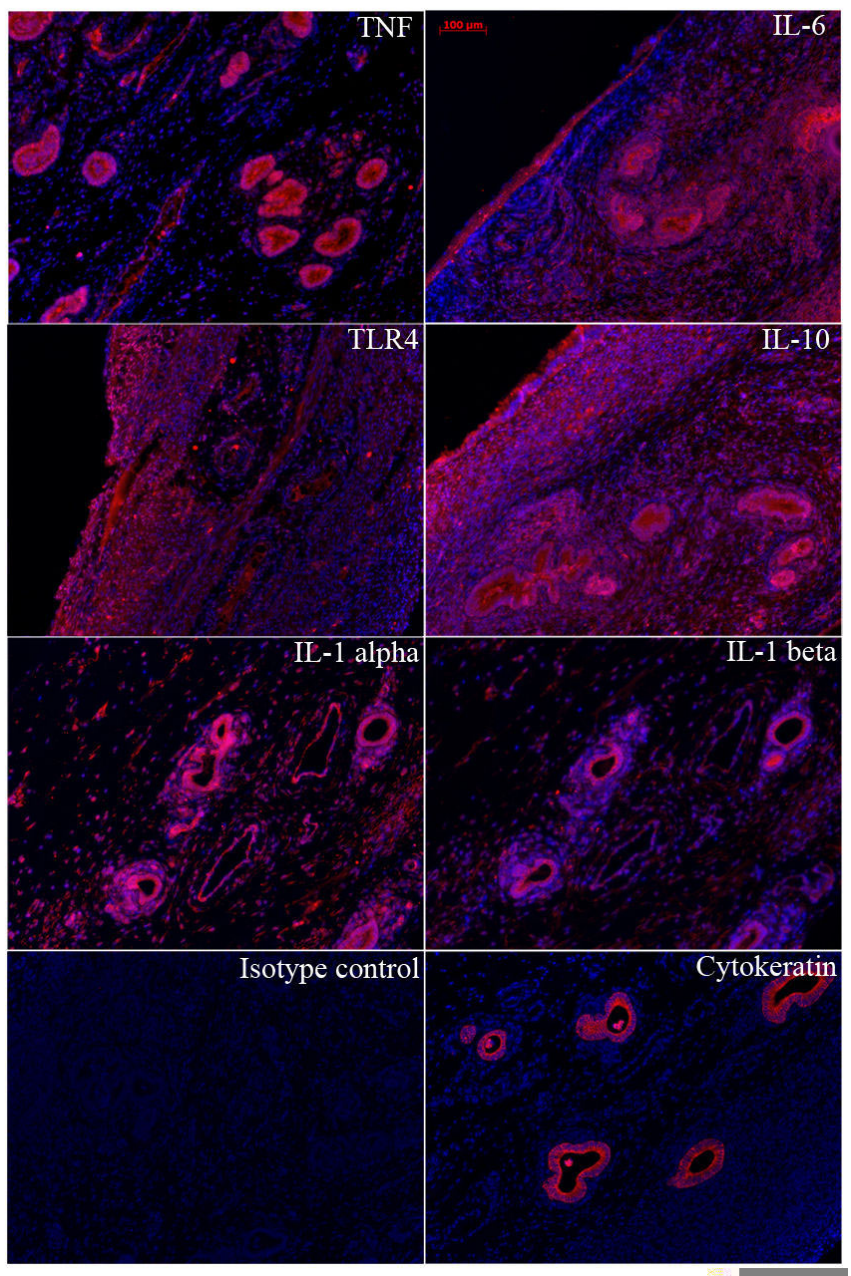
**Endometrial expression of TNF, IL-6, TLR4, IL-10, IL-1 alpha, IL-1 beta and cytokeratin protein**. Immunohistochemical localization of TNF, IL-6, TLR4, IL-10, IL-1 alpha, IL-1 beta and cytokeratin in the endometrium of postpartum cows. 5 μm sections of Formalin-fixed paraffin-embedded endometrial biopsies were examined using the primary antibodies described in Methods with Alexa Fluor 555 secondary antibodies, mounted with DAPI Vectashield. Representative images of TNF, IL-6, TLR4, IL-10, IL-1 alpha, IL-1 beta and cytokeratin immunoreactive protein and an isotype IgG control in endometrial tissue from animals week 1 post partum are shown. The scale bar is 100 μm.

Immuno-reactive protein was detected in the postpartum endometrium for TLR4, TNF, IL-6, IL-10, IL-1 alpha and IL-1 beta. (Fig. 8). The cytokines were particularly expressed by the glandular epithelium, although IL-6, IL-10 and IL-1 alpha were evident in the stroma as well. TLR4 was also expressed in the stroma as well as by epithelial cells.

## Discussion

Bacterial contamination of the uterine lumen is common in cattle following parturition [1-3]. Although all animals are exposed to similar levels of microbial contamination with bacteria from the environment, some animals successfully clear bacteria from the female genital tract and are fertile, while other animals have a persistent infection resulting in varying degrees of uterine disease and infertility. As expected, the mRNA expression of genes encoding pro-inflammatory mediators such as *NOS2 *and the cytokines *IL1A, IL1B*, and *IL6 *were higher in the endometrium during Period 1 than 2, irrespective of subsequent disease or fertility. Animals with clinical endometritis that were infertile, had higher levels of mRNA encoding *TLR4, IL1A, IL1B *and *IL1R2 *than fertile animals during Period 1. There was no evidence of down regulation of mRNA for the other *TLRs *responsible for detection of pathogens, or changes in the prostaglandin E_2 _receptors PTGER1 and PTGER2, or *TGFBR1 *that might limit the severity or duration of the inflammatory response in fertile animals. However, the infertile animals had higher ratios of expression of *IL1A *or *IL1B *to the anti-inflammatory cytokine *IL10*. Endometrial tissues also expressed TLR4, IL-1 alpha, IL-1 beta, and IL-10 protein. The data do not elucidate the underlying mechanism for the differences in endometritis between fertile and infertile animals, which could include genetic components, metabolic and other mediators not investigated, and the effects of particular strains of bacteria. However, we suggest that a dominant pro-inflammatory response during the first week post partum is associated with persistent endometritis and infertility.

A common bacterial contaminant of the uterine lumen in cattle is *E. coli *[1,2,22,24]. The *E. coli *pathogenic moiety, LPS, is recognised by the receptor complex TLR4/MD-2/CD14 [10,11], and endometrial cells express TLR4/MD-2/CD14 [5,7,12,27]. The endometrial cells respond to bacteria with the production of prostaglandins, particularly prostaglandin E_2_, and pro-inflammatory cytokines [5,7,28]. These data demonstrate an ability of endometrial cells to respond to bacteria in the absence of professional immune cells. Bacteria, other than *E. coli *were also isolated from the postpartum uterus and in the present study the endometrium expressed all the family of TLRs responsible for recognising pathogen associated molecular patterns. As well as the capacity of endometrial cells to respond to infection, both groups of animals had infiltration of professional immune cells as determined by *CD45 *expression, although there tended to be a higher level of expression of the pan-leukocyte marker *CD45 *in infertile animals. The heightened immune response during Period 1 than 2 was also associated with a higher level of expression of mRNA encoding *TLR4 *and *TLR2*, which recognise bacterial LPS and lipopeptides, respectively [10,11].

Immune mediators including IL-1, IL-6, interferon alpha, TNF and NOS2, play an important role in pathogen clearance [14], as well as several roles in reproduction [19-21,29]. However, their gene expression did not differ significantly between the animal groups, except for *IL1A *and *IL1B*. The roles of IL-1 in immunity are multiple, including the augmentation of lymphocyte responses and stimulation of acute-phase proteins [30]. Two isoforms of IL-1 exist, IL-1 alpha and IL-1 beta, and although their biological functions are similar, there are subtle differences between them (reviewed in reference [30]). IL-1 also plays a pivotal role in reproduction, demonstrating an involvement in ovulation as well as in oocyte maturation [31,32]. Consequently, we investigated the presence of mRNA encoding IL-1 in the endometrium of animals with disparate disease outcomes and found that during Period 1, infertile animals had higher levels of *IL1A *and *IL1B*, and their cognate *IL1R2*, compared with fertile animals. Importantly, responses to IL-1 occur at the femtomolar concentrations, and studies have shown low doses of IL-1 are protective against infection challenge of rodents while larger amounts of the cytokine are detrimental [30]. Furthermore, IL-1 also modulates endometrial prostaglandin secretion [21,32]. Taken together, the present results suggest that during Period 1 there is a heightened IL-1 response, which may be detrimental because animals with this elevated response failed to conceive despite a down-regulation in the level of mRNA in Period 2.

The control of pro-inflammatory responses to avoid excessive immune activation by bacteria, including the effects of IL-1, are dependent on anti-inflammatory mediators such as IL-10, transforming growth factor beta 1 and prostaglandin E_2 _[16,17,26]. The latter two molecules are abundant in the endometrium, although there is less information on IL-10. There was no difference in the level of expression of *IL10*, the transforming growth factor beta 1 receptor, *TGFBR1*, or the prostaglandin E_2 _receptor genes *PTGER2 *and *PTGER4 *between the groups of animals. Furthermore, there was no evidence of damping down of the inflammatory response by reduced expression of the TLRs, or changes in expression of *PTGER2, PTGER4 *or *TGFBR1*. However, the *IL1A *or *IL1B *to *IL10 *ratios were higher in the infertile than fertile animals during Period 1. In human endometrial stromal cells, IL-10 is able to inhibit the TNF-induced production of RANTES (regulated upon activation, normal T cell expressed and secreted) or IL-6 [33,34]. So, IL-10 may have a role to limit the pro-inflammatory response in the endometrium of postpartum cattle.

Although a proteomic investigation was not the aim of the present study, immunohistochemistry was used to confirm the presence of key proteins identified using quantitative PCR and explore their localisation in the endometrium. There was prominent expression of cytokines such as IL-1, IL-6, TNF, and IL-10 in the endometrial epithelium, as well as TLR4. This localisation may reflect that the epithelium is the first line of defence against pathogens [8]. Although the present study encompasses a limited number of animals it should provide a basis for more focussed studies to study the role of pro- and anti-inflammatory molecules in regulating the progression of endometritis.

## Conclusion

In conclusion, animals that had persistent endometritis and were infertile had a greater pro-inflammatory response to bacterial infection during the first week post partum, than fertile animals. The expression of innate immune receptors did not differ between the fertile and infertile groups, except for *TLR4*, which is required for detection of LPS. The key difference between the groups appeared to be higher ratios of the mRNA for the pro-inflammatory cytokines *IL1A *or *IL1B *to the anti-inflammatory cytokine *IL10*. Further work is required to determine the mechanisms underlying the differences in gene expression between infertile and fertile postpartum cattle, including the role of genotype, metabolic factors and strains of bacteria.

## Competing interests

IMS, ROG, JOW, CEB, SH and JC hold research funding under a Department for Environment Food and Rural Affairs (DEFRA) LINK award from Pfizer Animal Health and the Biotechnology and Biological Sciences Research Council (BBSRC; Grant No. F005121). LG works for Pfizer Animal Health. The remaining authors declare that they have no competing interests.

## Authors' contributions

IMS, ROG, JOW, CEB, SH and LG were awarded the grants to fund the work, devised experiments, analysed the data and wrote the manuscript. SH and STL performed the molecular biology. JC performed the immunohistochemistry and contributed to the manuscript. ROG and NRS conducted the animal study and collected the tissue samples. All authors read and approved the manuscript.
